# Relationship of footwear comfort, selected size, and lower leg overuse injuries among infantry soldiers

**DOI:** 10.1186/s12891-021-04839-9

**Published:** 2021-11-15

**Authors:** Darja Nesterovica, Normunds Vaivads, Ainars Stepens

**Affiliations:** 1 Military Medicine Research and Study Centre, Rīga Stradiņš University, 14 Baložu Street, Riga, LV-1048 Latvia; 2 Latvian National Armed Forces Joint Headquarters Medical Service, Kadaga, 2103 Latvia

**Keywords:** Military personnel, Footwear comfort, Overuse injuries, Military boot

## Abstract

**Background:**

High rates of musculoskeletal injuries such as plantar fasciitis and stress fractures have been observed among physically active military personnel. During service time, infantry soldiers use issued boots daily that should fit well and provide comfort to prevent injuries and decrease lower extremity pain effectively. The association of military boot comfort with overuse injuries remains unclear. This study investigates the relationship between the chosen military boot size, perceived boot comfort and lower leg overuse injury.

**Methods:**

During the cross-sectional study, 227 (males, n = 213; females, n = 14) active-duty infantry soldiers at a mean age of 29.5 years old, and with an average service time of 7.2 years were assessed for a history of overuse injury, footprint length, appropriate shoe size, and footwear comfort. Males with a history of overuse injury (n = 32) and non-injured age-matched controls (n = 34) were selected for detailed testing and establishing the possible relationship between footwear comfort and lower leg overuse injury.

**Results:**

No relationship was found between footwear comfort and a history of lower leg overuse injury. *N =* 38 (57.6%) of study subjects were wearing an inappropriate shoe size daily. Inappropriate shoe size usage affected footwear comfort ratings significantly.

**Conclusions:**

Study results showed that improper boot size was significantly related to comfort ratings but was not associated with a history of lower leg overuse injury.

**Supplementary Information:**

The online version contains supplementary material available at 10.1186/s12891-021-04839-9.

## Background

Most military personnel require high physical demands during service time. It has been reported that 41–67% of sustained injuries in the military affect the lower extremities [1–3]. Typical injuries associated with physical training and prolonged load carriage are cumulative micro-traumatic lower extremity overuse injuries [4]. Injuries such as stress fractures, shin splints, patellofemoral pain, plantar fasciitis, and Achilles tendinopathy reduce military readiness and could even be a reason for medical discharge [5, 6]. This study explores military boot comfort and its relationship with musculoskeletal overuse injury in detail.

During training or actual combat scenarios, military personnel use military boots that protect the shank and foot from environmental hazards such as irregular and uneven terrain. Foot health and footwear comfort are crucial for the military readiness of infantry soldiers. Shock absorbance and stability on uneven terrain are also very important military footwear features. Footwear shock-absorbance study results among Israeli infantry recruits showed that soldiers who used basketball shoes during basic training had a lower incidence of overuse injuries of the foot (18%) than those who wore infantry boots (34%). The authors of the study concluded that the basketball shoes’ shock attenuation reduced foot overuse injuries, but not injuries at other lower extremity locations [7]. Other studies showed that military footwear specifically made for prolonged standing and marching, adverse weather conditions, and with a proper fit may effectively prevent injuries and decrease lower extremity pain [8, 9].

Footwear comfort is a complex combination of several factors including good fitting, internal temperature, humidity environment, plantar pressure distribution, and ground impact force [10–12]. As reported by a recent systematic review, a large proportion of the population wears ill-fitting shoes that contribute to foot pain and foot disorders [13]. Research evaluating shoe sizing on the subjective fit and comfort of shoes is encouraged [14]. Pressure-induced skin lesions and toenail problems are clinical effects of poor-fitting or uncomfortable footwear observed in the general population, especially those with chronic foot disorders [15, 16]. Footwear comfort has been proposed as an important factor for all movement-related lower extremity injuries [17, 18]. Associations of chronic foot disorders (e.g., pes planus, hallux valgus) and acute injuries (ankle fracture or sprain) with boot usage among military populations, as well as military boot functional needs were established previously [19, 20]. This study compares the used infantry boot size (subjective fit) with correct fit according to bare footprint length among infantry soldiers with and without a history of lower extremity overuse injury.

## Methods

**Fig. 1:**
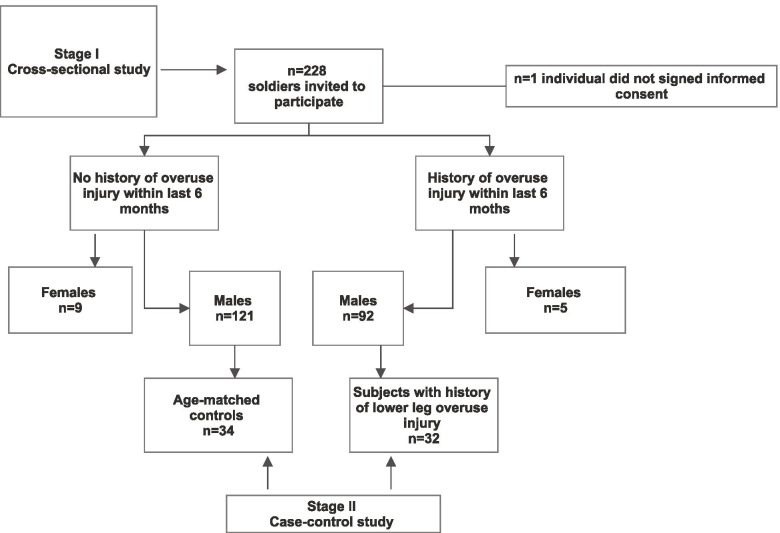
Flowchart of the study design

We carried out a study designed in two stages: stage I - cross-sectional study and stage II case-control study. Flow chart of the study design is seen in Fig. 1.

In 12 consecutive interview sessions total, 228 (16%) of all active-duty infantry soldiers of Latvian Land Forces (males, *n* = 214; females, *n* = 14) were invited to participate in our study during the annual medical check-up at the Latvian National Army Logistic Command Military Medical Support Centre. Participation was voluntary, and the study results did not change the annual medical check-up results. Before entering the study, written informed consent was provided for each potential study participant; one person did not sign the informed consent, and according to the protocol, 227 infantry soldiers were selected for further activities. Their mean age was 29.5 ± 7.1 years old (range 20–49 years), service time 7.2 ± 6.4 years (range 0.5–25 years). Study population characteristics are shown in Table 1.

**Table 1:** Cross-sectional study population characteristics

	Total (*n* = 227)	Males (n = 213)	Females (*n* = 14)
Age, years (SD)^a^	29.5 (7.2)	29.4 (7.0)	32.1 (8.3)
Service time, years (SD)	7.2 (6.4)	7.1 (6.4)	8.3 (6.5)
History of total lower extremity overuse injury, % (n)	42.7 (*n* = 97)	43.2 (*n* = 92)	35.7 (*n* = 5)
History of lower leg and foot overuse injury, % (n)	15.0 (n = 34)	15.0 (n = 32)	14.3 (n = 2)
Foot blisters after long marching, % (n)	46.3 (*n* = 105)	46.5 (*n* = 99)	42.9 (n = 6)
Usage of foot orthotics, % (n)	4.9 (*n* = 11)	4.7 (n = 10)	7.1 (n = 1)

^a^Standard deviation (SD) is given in brackets

The musculoskeletal injury was considered if soldier either reported or had a medical record of injury, which did not allow participation in at least one activity during the last 6 months of service. Musculoskeletal injuries were classified into two groups: acute and overuse injuries and the coding was performed by the interviewer (DN). The acute injury was defined as an injury due to blunt, crushing, penetrating trauma. Acute injuries are strains, sprains, ligament ruptures, fractures [excluding stress fractures] and were classified by ICD-10 (International Classification of Diseases, Tenth Revision) codes S00-T32 [21]. Overuse injuries were defined as injuries caused by repetitive or forceful tasks resulting from repeated overstretching or overloading [22]. Injuries such as anterior or posterior tibial syndrome (ICD-10 code M76.8), plantar fasciitis (M72.2), Achilles tendonitis (or bursitis, M76.6), peroneal tendinitis (M76.7), and stress fractures (M84.3) were classified as overuse injuries. For both types of injury, body regions were classified in the same manner as in the Barell injury matrix [23].

For this study, we have prepared a military comfort assessment tool according to the previously used methodology [24]. A visual analogue scale with a ten-centimetre length was used to rate the footwear comfort for six dimensions: overall comfort, forefoot cushioning, arch cushioning, heel cushioning, arch support, heel support, according to a previously used method. The left end was labelled as ‘not comfortable’ (0) and the right end was labelled as ‘best comfort’ (10). Example is shown in Additional file 1.

For the second stage of our study, we have invited all 32 (14%) subjects with a history of the lower leg, ankle, and foot overuse injury and 34 (15%) age-matched non-injured subjects for more detailed testing. Visual inspection of the skin and nails of the foot and bare footprint length were additionally assessed. The presence of blisters, corns, or calluses, as well as ingrown toenails and subungual haematoma, were documented according to the classification by Carr&Cropley [25]. Characteristics of the case-control study population are shown in Table 2.

**Table 2:** Case-control study population characteristics

	Total (n = 66)	Subjects with prior OI^**a**^ (n = 32)	Non-injured subjects (n = 34)	***P*** value^**b**^
Age, years	29.7 (5.5)	29.0 (5.7)	30.5 (5.3)	0.12
Height, m (SD^c^)	1.81 (0.13)	1.81 (0.13)	1.81 (0.13)	0.96
Weight, kg (SD)	81.3 (12.9)	81.3 (13.3)	81.2 (12.6)	0.96
Foot length, mm (SD)	274 (13)	275 (13)	273 (13)	0.19
**Usage of foot orthotics**, % (n)	(*n* = 4)	12.5 (n = 4)	0	**0.04**
Foot blisters after long marching, % (n)	57. 6 (n = 38)	53.1 (*n* = 17)	61.8 (n = 21)	0.16
Foot skin lesions, % (n)	(n = 14)	(n = 6)	(*n* = 8)	0.58
**Toenail problems**, % (n)	(n = 18)	(n = 14)	(n = 4)	**0.01**

^a^OI – overuse injury. ^b^One-way ANOVA test results; significant results are marked in bold. ^c^Standard deviation (SD) is given in brackets

For footprint length assessment, participants were asked to stand in a relaxed manner on a pressure platform (2 m × 0.4 m × 0.02 m, RSscan International, Belgium). Platform calibration was performed before each measurement. Plantar pressure analysis software (Footscan® v.7.11, RSscan International) was used to detect the precise footprint length in millimetres. Footscan® pressure plate has shown good repeatability and is commonly used in foot pressure and foot area data assessment [26, 27]. To detect the correct shoe size, bare footprint length was converted to shoe size using the metric footwear sizing — Mondopoint system [28]. In the case of footprint length difference, the longer foot was chosen to analyse footwear sizing. A comparison of the used self-selected shoe size with a correct shoe size was made according to the bare footprint length. The correct fit was defined if the self-selected footwear size matched the Mondopoint sizing.

The size of issued military boots was self-selected based on soldier’s previous shoe fitting experience; each size has only one width and half-sizes have not been provided. The footprint width was not analysed. Given that Latvia’s average annual air temperature is + 5.9 °C [29], and for most of the year soldiers use boots for hot weather conditions, we assessed the footwear comfort rating for this type of issued infantry boot only.

Statistical analysis was performed using the SPSS 22.0 software package (Statistical Package for the Social Sciences). Data were explored for distribution; normality was investigated using the Kolmogorov-Smirnov test. If data did not meet normal distribution assumptions, non-parametric tests were applied. Quantitative variables are presented as means with standard deviation; categorical variables are presented as frequencies if not stated otherwise. The study sample was defined as an “availability sample”. Sample size calculations were based on one-year musculoskeletal lower extremity injury among Latvian Land Forces (12.4%) and performed using the open-source calculator (OpenEpi, Open Source Statistics for Public Health) [30, 31]. The significance level was set to *p* < 0.05 (two-tailed), and statistical power was set to 0.9.

## Results

### Footwear comfort rating

Footwear comfort rating was assessed for all study participants (*n* = 227). Differences in footwear comfort rating between gender groups were independent of the previous history of overuse injury. The highest overall footwear comfort rating was 6.7 in the non-injured males group. The lowest rating of 5.2 was observed for the heel cushioning among the non-injured females group. Mean footwear comfort ratings among females were lower across all dimensions, but the difference with the male group was not statistically significant (see Table 3).

**Table 3:** Mean military footwear comfort ratings

	Males (n = 213)		Females (*n* = 14)	*P*-value^c^	
	With prior OI^a^ (n = 92)	Non-injured (*n* = 121)	With prior OI^a^ (n = 5)	Non-injured (n = 9)	
Overall comfort	6.3 (1.8) ^b^	6.7 (1.7)	5.6 (2.1)	6.1 (2.2)	0.16
Forefoot cushioning	6.0 (1.9)	6.4 (1.8)	5.6 (1.7)	5.7 (2.0)	0.12
Arch cushioning	6.1 (1.8)	6.2 (2.0)	5.6 (1.8)	6.1 (1.7)	0.67
Heel cushioning	6.2 (1.8)	6.2 (2.0)	5.6 (1.3)	5.2 (2.0)	0.84
Arch support	6.0 (1.9)	6.4 (1.9)	6.0 (1.7)	5.7 (1.9)	0.19
Heel support	6.2 (1.9)	6.7 (1.8)	5.8 (1.6)	6.0 (2.4)	0.05

^a^OI – overuse injury; ^b^ Standard deviations are given in brackets; ^c^ One-way ANOVA test results comparing injured and non-injured groups

### Footwear sizing analysis

In total, *n* = 66 male subjects were additionally tested to assess the relationship between footwear comfort and lower leg overuse injury. For the additionally tested group, self-selected military footwear sizes were converted to mm (millimetres) using the Mondopoint system and then compared with the footprint length measurement from the Footscan® software. As a result, 57.6% (*n* = 38) of all study subjects daily were wearing an inappropriate shoe size: 30.3% among subjects with a history of overuse injury (*n* = 20) and 27.3% among subjects without a history of overuse injury (*n* = 18). Only six subjects wore bigger shoe sizes, and others (*n* = 31) used a smaller shoe size than would be recommended according to their foot measurement. Self-selected shoe sizes were statistically significantly different among groups (*p* = 0.04). The median footprint length difference between the left and right sides was 1 mm (range 0–5 mm). See Table 4 for details.

**Table 4:** Military footwear sizing preferences

	Total (n = 66)	Subjects with prior OI^**a**^ (n = 32)	Non-injured subjects (n = 34)	P value^**b**^
**Self-selected EU**^**d**^ **shoe size**, (SD^c^**)**	43 (1.5)	43.5 (1.6)	43 (1.4)	**0.04**
**Measured EU shoe size**, (SD)	43.6 (1.6)	43.9 (1.6)	43.4 (1.5)	**< 0.01**
Suitable shoe size usage, % (n)	42.4 (n = 28)	37.5 (n = 12)	47.1 (n = 16)	0.16
Inappropriate shoe size usage, % (n)	57.6 (n = 38)	62.5 (n = 20)	52.9 (n = 18)	

^a^OI – overuse injury. ^b^Chi-square test results; significant results are marked in bold. ^c^Standard deviation (SD) is given in brackets

^d^EU – European shoe size

### Lower extremity overuse injury and comfort rating

Subjects who wore the wrong shoe size in both (injured and non-injured) groups showed lower military footwear perceived comfort ratings across all dimensions, independent of previous lower extremity overuse injury. For most of the comfort dimensions, the difference between injured and non-injured groups was statistically significant. Detailed results are shown in Table 5.

**Table 5:** Military footwear comfort rating comparison among study subjects

	Subjects wearing inappropriate shoe sizes (*n* = 38)		Subjects wearing suitable shoe sizes (*n* = 28)		χ^2^(1)	P value^†^
	With prior OI (*n* = 20)	Non-injured (*n* = 18)	With prior OI (*n* = 12)	Non-injured (*n* = 16)		
Overall comfort	6.69 (1.22)	6.91 (1.11)	7.29 (1.04)	7.28 (1.33)	5.23	**0.02**
Forefoot cushioning	6.24 (1.57)	6.18 (1.78)	7.00 (0.98)	6.59 (1.72)	4.17	**0.04**
Arch cushioning	6.24 (1.57)	6.15 (1.79)	6.88 (1.36)	6.53 (2.00)	3.61	**0.06**
Heel cushioning	6.29 (1.38)	6.26 (1.52)	6.92 (1.38)	6.66 (1.66)	5.06	**0.03**
Arch support	5.90 (1.79)	6.15 (1.74)	6.75 (1.59)	6.63 (1.88)	4.38	**0.04**
Heel support	6.38 (1.61)	6.47 (1.58)	7.58 (1.02)	7.19 (1.18)	11.07	**< 0.01**

OI – overuse injury. ^†^Kruskal Wallis test results; standard deviation is given in brackets. Significant results are marked in bold

## Discussion

To the author’s knowledge, this is the first attempt to systematically evaluate perceived footwear comfort for different boot dimensions in a relationship with previous foot overuse injury among infantry soldiers. The present study assessed military boot comfort ratings and footwear fit among infantry soldiers with and without a history of lower extremity overuse injury. However, the overuse injury definition used widely is not uniform, we used the definition that emphasises a mechanism of gradual onset and underlying pathogenesis of repetitive microtrauma as was recommended by Roos et al. [32] Previous military footwear research performed in 1976 focused on different lower extremity disorders, both acute (ankle fractures) and overuse injuries (heel contusions, toe paresthesia, and retrocalcaneal bursitis), and military boot comfort data for different boot dimensions remained unknown [19, 20]. According to Dijksma et al. findings of previous footwear research among military populations may no longer apply due to the design of military boots evolving [33]. Current military boot design should contribute to better perceived comfort and a standardised military footwear comfort evaluation tool is needed.

Footwear comfort measures are difficult to compare with other studies due to methodological differences. Perceived comfort perception in our study was measured using a visual analogue scale, not only for overall comfort but also for cushioning and supporting different parts of the foot [24]. Muniz et al. only reported overall footwear comfort among Brazilian army recruits that varied from 5.5 to 7.7 points, with higher comfort provided by softer midsole and lower boot weight [34]. Paisis et al. investigated perceived comfort among the Greek army, and study results showed that participants also preferred walking with the lightest weight boot. It has been reported that reduced weight, increased stiffness, and the construction of military boots could be beneficial for higher footwear comfort [35]. Types of military footwear materials, shock-absorbing possibilities, microclimate features, footwear width, and footwear weight, as well as gait kinematics, were not assessed in our study.

Footwear sizes in the Latvian Land Forces are self-selected by the soldier. Footwear sizes vary among producers, and the soldier’s choice of footwear size is based on previous experience, which can be wrong. Study findings conducted among infantry of Canadian Land Forces showed that personnel footwear was not appropriately fitted according to foot length and width [36].

We compared self-selected footwear sizes with recommended footwear sizes (based on footprint length). We used a universal Mondopoint footwear size measurement system for size conversion, which is performed on a statistically constructed human foot and uses foot length in millimetres. Our study findings showed that 56% of study participants wore inappropriate shoe sizes, and these results are consistent with the previously mentioned study [36]. Wearing incorrectly sized footwear is a common problem, and it has been associated with foot pain and foot disorder [13]. The shoe’s fit has been associated with skin disorders of the foot such as corns and calluses. In our study, foot skin disorders were not prevalent among both study groups, and recently it has been proposed that corns and calluses could indicate the asymmetrical behaviour of the lower limbs during gait [37]. Toenail disorders, which could result from the tight toe box of footwear [25], were more prevalent among subjects with prior overuse injury who used an inappropriate shoe size. Highly rated footwear comfort is possible if the proper fit is provided, and our study results show moderately low comfort ratings.

Study subjects who used inappropriate shoe sizes showed statistically significantly lower military footwear perceived comfort ratings across all dimensions, and these results are partly consistent with previous findings. It has been reported that inappropriate shoe fit could lead to discomfort and contribute to lower extremity overuse injury due to gait adaptations [38]. However, the complexity of what makes the appropriately fitted shoe more comfortable, and the impact of shoe comfort on gait and pathology is not yet well understood [39].

Our study results found no relationship between footwear comfort ratings and lower extremity injury history. Grier et al. have identified that better cushioned footwear did not lower injury incidence, although poor footwear fit and cushioning were associated with foot pain and discomfort. Our study results showed that subjects wearing the wrong shoe size reported lower footwear comfort ratings. To potentially increase footwear cushioning and comfort shock-absorbing insoles have been recommended [8, 40]. Prefabricated foot orthoses were found to be effective in preventing lower limb overuse injuries [41].

Current study findings should be considered in the context of study limitations. The cross-sectional study design is a limitation due to the inability to establish causal sequences and recall bias of injury history. Although the study population is relatively small, it is representative (*n* = 227) and considerably larger than calculated sample size (*n* = 150). Grouping of the case-control study also depends on participant honesty, and it has been reported that approximately half of the injuries among military populations are not usually reported to medical personnel [42]. We believe that answers to the interviewer were honest because soldiers were informed that the study results would not affect the medical annual check-up status. Also, comfort ratings could influence the fact that only one type of infantry boot (for hot weather conditions) was assessed. Additionally, including foot width could provide more detailed comfort ratings, but since it did not impact boot size measurements, it was not included in the analysis. We did not check if the same boot pair was used for the last 6 months; however, all soldiers of the Land Forces of Latvia use the same boot model, and in any case, comfort ratings were provided for the same boot model. Given that perceived footwear comfort rating could change during physical activity due to fatigue [43], our study participants rated footwear comfort during a day-off to avoid the possible skewing of comfort data. The use of Footscan® software for foot length measurement was selected as reliable since digital footprint measurement for foot length assessment was found to be similar to a 3D (three-dimensional) foot scan [44]. Despite these limitations, the strength of this research is that it comes from a relatively homogeneous population and helps to gain a deeper understanding of military footwear fit and comfort by comparing previously injured and non-injured infantry soldiers groups.

According to our study, proper fit is an essential factor that leads to more comfortable military footwear usage. It is recommended to issue adequate military footwear size according to foot dimension measurement using a Brannock device or 3D foot scan to provide better footwear comfort. The findings of this study can also provide valuable information on footwear comfort to other users of work boots.

## Conclusions

To the authors’ knowledge, this is the first study of subjective infantry boot fit and comfort among infantry soldiers considering a history of lower extremity overuse injury. Study results showed that inappropriate infantry boot size significantly affects footwear comfort ratings. History of previous lower extremity overuse injury was not related to either shoe size selection or footwear comfort ratings. Based on our study results, we recommend footprint length assessment for proper footwear size selection.

## Supplementary Information

**Additional file 1.** “Military_boot_comfort_tool.pdf”, example of visual analogue scale used for footwear comfort assessment.

## Data Availability

Datasets analysed in this study are not publicly available because the Latvian National Army Logistic Command Military Medical Support Centre did not permit data sharing. Request to access the datasets should be directed to the corresponding author.

## Abbreviations

ICD-10: International Classification of Diseases Tenth Revision
SPSS: Statistical Package for the Social Sciences
mm: millimetres
3D: three-dimensional
