# Supplementary material for: Relationship of footwear comfort, selected size, and lower leg overuse injuries among infantry soldiers
Source: BMC Musculoskelet Disord. 2021 Nov 15;22:952. doi: 10.1186/s12891-021-04839-9 (PMC8594192; doi:10.1186/s12891-021-04839-9)
Supplement: Supplementary file 1 — Additional file 1. “Military_boot_comfort_tool.pdf”, example of visual analogue scale used for footwear comfort assessment. [file 12891_2021_4839_MOESM1_ESM.pdf]

Subject ID_________

Military boot comfort assesment tool

Age (years): __________

Shoe size (EU): ________

With regard to your military boot (for hot weather conditions), please rate the following by placing a single vertical line on the scale:

1. **Overall military boot comfort**

Not comfortable at all ______________________________ Most comfort imaginable

2. **Forefoot cushioning**

Not comfortable at all ______________________________ Most comfort imaginable

3. **Arch cushioning**

Not comfortable at all ______________________________ Most comfort imaginable

4. **Heel cushioning**

Not comfortable at all ______________________________ Most comfort imaginable

5. **Arch support**

Not comfortable at all ______________________________ Most comfort imaginable

6. **Heel support**

Not comfortable at all ______________________________ Most comfort imaginable

Thank you for your answers!
